# Young women with (pre)malignant cervical disease in northern Netherlands: what characterises them – A population-based cohort study

**DOI:** 10.1186/s12905-026-04598-6

**Published:** 2026-06-11

**Authors:** Marjolein Dieleman, Karin M. Vermeulen, Albert G. Siebers, Bea A. Wisman, Ed Schuuring, Martha D. Esajas, Daan Brandenbarg, Saskia W. M. C. Maass, Geertruida H. de Bock

**Affiliations:** 1 Department of Epidemiology, University of Groningen, University Medical Centre Groningen, Hanzeplein 1, Groningen, 9700 RB the Netherlands; 2 Palga, Dutch nationwide pathology databank, De Bouw 123, Houten, 3991 SZ the Netherlands; 3 Department of Gynaecologic Oncology, University of Groningen, University Medical Centre Groningen, Hanzeplein 1, Groningen, 9700 RB the Netherlands; 4 Department of Pathology, University of Groningen, University Medical Centre Groningen, Hanzeplein 1, Groningen, 9700 RB the Netherlands; 5 Department of Obstetrics and Gynaecology, University of Groningen, University Medical Centre Groningen, Hanzeplein 1, Groningen, 9700 RB The Netherlands; 6 Department of Primary and Long-Term Care, Cure and Care in the Community Context (FOUR-C) research programme, University of Groningen, University Medical Centre Groningen, Hanzeplein 1, Groningen, 9700 RB The Netherlands

**Keywords:** Uterine Cervical Neoplasms, Cervical Intraepithelial Neoplasia, Socio Demographics, Young Adult, General Practice

## Abstract

**Background:**

Little is known on (pre)malignant cervical disease in women below the age of population-based screening (PBS). The aim of this study was to evaluate the occurrence of (pre)malignant cervical disease and related sociodemographic characteristics in women who are tested and/or referred by the GP before reaching the eligible age (30 years) for participation in the first round of PBS for cervical cancer in the northern Netherlands.

**Methods:**

A linkage was performed between the Lifelines cohort (*N* = 88439 women) and the Dutch nationwide pathology databank (Palga). Data on cervical test records and sociodemographics such as socio-economic status and smoking status were retrieved and analysed.

**Results:**

A cervical test record before the first round of PBS was reported for 6301 women. Cervical intra-epithelial neoplasia grade 2 or higher (CIN2+) was diagnosed in 198 (3.1%) women. CIN2 + was associated with low educational level (adjusted odds ratio (aOR) 1.7, 95% confidence interval (CI) 1.1–2.4), ex-smoking (aOR 1.8, 95%CI 1.2–2.9) and current smoking (aOR 2.7, 95%CI 1.9–3.8).

**Conclusions:**

Within women tested and/or referred by the GP before the eligible age for the Dutch cervical cancer PBS, 3.1% was diagnosed with CIN2+. Higher odds of CIN2 + for women with a low educational level and women who (used to) smoke should be taken along in clinical decision making towards testing and referral.

**Supplementary Information:**

The online version contains supplementary material available at 10.1186/s12905-026-04598-6.

## Background

The worldwide burden of cervical cancer remains high, with an estimated 661,000 new cases and 348,000 deaths in 2022 [1]. Infection with high-risk human papillomavirus (hrHPV) types almost always underlies the usually slow process from healthy to malignant tissue [2]. During this process, premalignant lesions can be detected and effectively treated, making the disease a suitable target for population-based screening (PBS). The starting age of PBS varies internationally. For example, in Denmark, women are first invited at age 23, while in the United Kingdom and in France, the PBS starts at age 25 [3]. The World Health Organization (WHO) recommends starting PBS at the age of 30 [4]. In the Netherlands, women between the ages of 30 and 60 years are invited to PBS every five to ten years, for a primary hrHPV-test and subsequent cytology triage in case of hrHPV positivity [5].

Dutch women under the age of 30 are only tested for (pre)malignant cervical disease if they present with symptoms, typically during a visit to primary care, as the Dutch general practitioners (GPs) have a gatekeeper function to the health care system. Outside of the PBS, about 40 patients per year per average general practice present with symptoms suggestive of the presence of (pre)malignant cervical disease in all age groups, such as postcoital or abnormal bleeding [6, 7]. However, as the incidence of cervical cancer in the Netherlands is relatively low, there will only be about 0.4 diagnoses of cervical cancer per year per average general practice, including the diagnoses detected through the PBS [6]. To make informed decisions about testing and referral of women with symptoms suggestive of the presence of (pre)malignant cervical diseases, GPs need to distinguish between women at higher and lower risk, especially in those not (yet) eligible for regular testing through the PBS. Knowledge of the occurrence of (pre)malignant cervical disease and possibly associated sociodemographic characteristics can be of essential aid in this. Among all age groups, it is known that behaviour and lifestyle can contribute to the risk of developing cervical cancer. For example, low age at first sexual intercourse, smoking, and long-term use of oral contraception are known to increase the risk of (pre)malignant cervical disease [7–9]. However, the association between these sociodemographic characteristics and (pre)malignant cervical disease for women younger than the eligible age for the PBS has hardly been investigated.

The aim of this study was to evaluate the occurrence of (pre)malignant cervical disease and associated sociodemographic characteristics in women who are tested and/or referred by the GP before reaching the eligible age for participation in the first round of PBS for cervical cancer in the northern Netherlands.

## Methods

### Context

This retrospective, longitudinal population-based cohort study was conducted in the north of the Netherlands. Current Dutch guidelines advise cervical cytology for women presenting with the following symptoms: abnormal fluor without clear cause, postcoital bleeding, intermenstrual bleeding, postmenopausal bleeding or abnormalities of the cervix encountered during physical examination [10, 11].

### Dataset

The analyses were conducted using data from Lifelines and Palga. Lifelines is a multi-disciplinary prospective population-based cohort study examining in a unique three-generation design the health and health-related behaviours of 167,729 persons living in the north of the Netherlands. It employs a broad range of investigative procedures in assessing the biomedical, socio-demographic, behavioural, physical and psychological factors which contribute to the health and disease of the general population, with a special focus on multi-morbidity and complex genetics [12, 13]. This data was linked to the Dutch nationwide pathology databank (Palga), in which all pathology records including data from the PBS are registered with nationwide coverage [14]. Data from both Lifelines and Palga included individuals of all ages.

### Primary outcome

The primary outcome was the absolute number of clinically relevant (pre)malignant cervical disease in women younger than the eligible age for the first round of PBS for cervical cancer (hereafter: women below PBS age), presenting with symptoms suggestive of the presence of (pre)malignant cervical disease. Clinically relevant (pre)malignant cervical disease was defined as histological diagnosis of cervical intraepithelial neoplasia grade 2 (CIN2), CIN3 or cervical carcinoma; hereafter “CIN2+”, as CIN2 generally represents the threshold at which treatment is recommended [15].

### Included participants

Women were included in the analysis for the primary outcome if they had a cervical test record in Palga registered under the age of 30. These tests would only have been performed for medical indication. Records obtained through or after PBS participation were excluded, also if this participation was at age 29, which was possible in a selected time period due to the timing of invitations from the PBS.

### Selection and definition of co-variables

Lifelines data was obtained through baseline questionnaires sent between 2006 and 2014 and multiple follow-up questionnaires over 10 years, incorporating both standardized instruments and study-specific questionnaires that have been described previously [13]. Factors associated with an increased risk of CIN2+, as identified in the literature and for which data were available in the Lifelines database, were selected [7–9]. The following was retrieved from the baseline questionnaires: (1) age and year, (2) income and (3) educational level as proxies for socio-economic status, (4) marital status, (5) hormonal contraceptive use as a proxy for oral contraceptive use, (6) number of pregnancies, (7) age at first child and (8) number of childbirths under the age of 30. From smoking data combined from multiple time points, (9) smoking status was derived. Since data on hrHPV vaccination status was unavailable, its potential influence was inferred from the number of women in eligible birth cohorts (1993 onwards). The following data from cervical test records was retrieved from the Palga database from the start of the registry in 1999 up until September 2021: (1) age (2) result or diagnosis based on cytology and/or histology and (3) performer. For other definitions of categories for categorical variables, see Table 1. Lastly, within the same cohort, a brief analysis was conducted on CIN2 + in women around the time of their first screening round (ages 29–34), to provide a rough indication of potentially undiagnosed (pre)malignant cervical disease below PBS age.

### Statistical methods

Sociodemographic characteristics of included women were described, overall and stratified for those with and without a diagnosis of CIN2+. Means and standard deviations (SD) and medians and interquartile ranges (IQR) were respectively used for normally and non-normally distributed continuous variables. The number of women with at least one test, at least one abnormal test result and at least one diagnosis of CIN2 + was plotted by age group. Odds ratios (OR) and 95% confidence intervals (95%CI) were calculated by logistic regression analyses performed separately for each sociodemographic characteristic, adjusted for age and year of completing the Lifelines questionnaire. A first sensitivity analysis included only women who completed the Lifelines questionnaire prior to CIN2 + diagnosis, to investigate possible impact of behavioural change after this diagnosis. A second sensitivity analysis only included women with CIN3 + lesions, defined as CIN3 or cervical carcinoma, as this might be considered to be more clinically relevant as compared to CIN2 [16]. P-values below 0.05 were considered statistically significant. Statistical analyses were performed in SPSS Version 28.

## Results

### Selection of women below population-based screening age

**Fig. 1:**
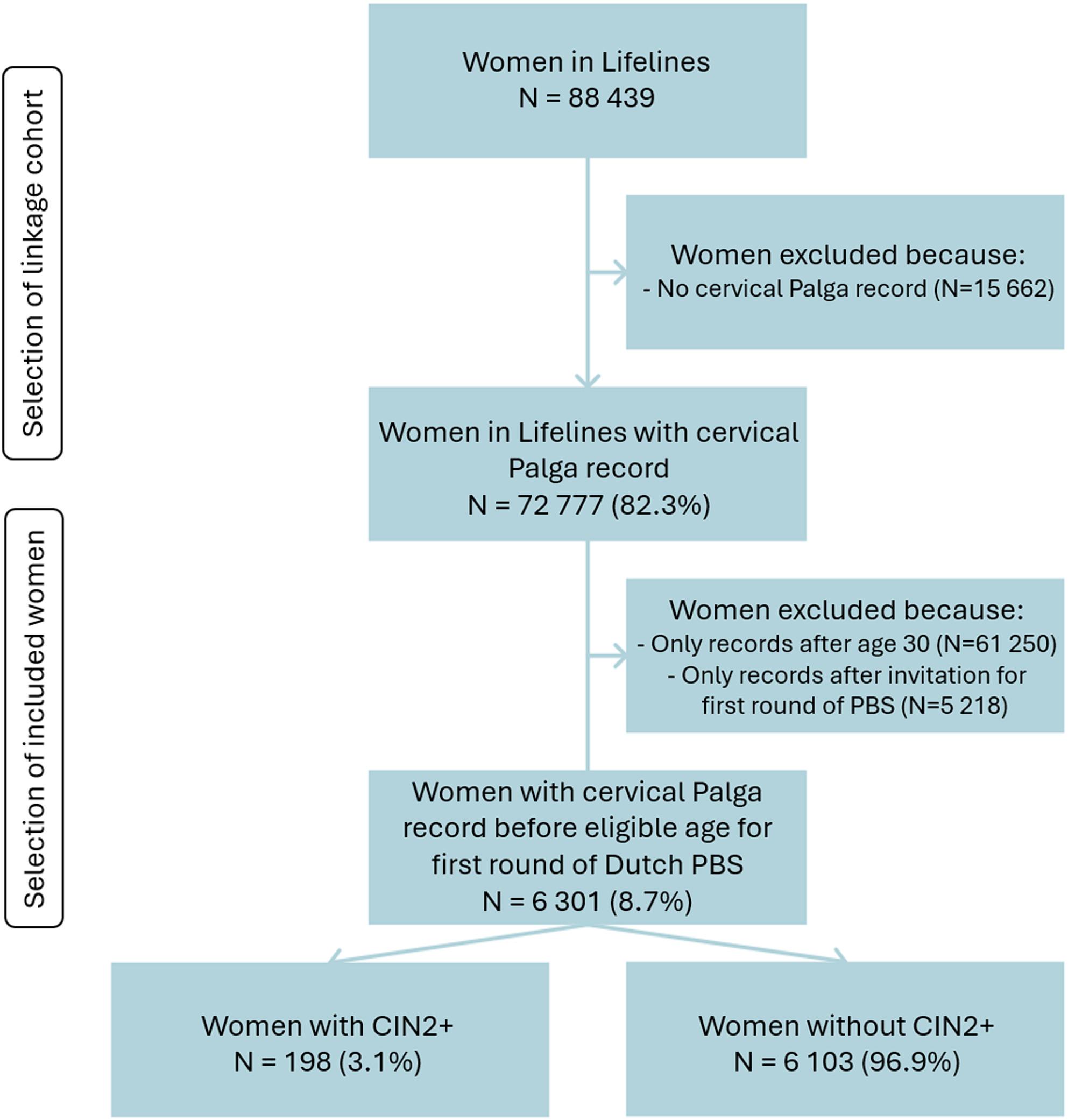
Included participants

In total, 88,439 women were included in Lifelines, of whom 72,777 (82.3%) had a cervical test record in Palga (see Fig. 1). Of these women, 6301 (8.7%) had a cervical test record below the PBS age. In this group, 198 women (3.1%) had one or more records in Palga reporting a histological diagnosis of CIN2+.

### Sociodemographic characteristics

As compared to women without a diagnosis of CIN2+, the women with a diagnosis of CIN2 + were younger when completing the Lifelines baseline questionnaire, had a lower income and lower educational level, were more frequently single, had a lower median number of pregnancies, had given childbirth less frequently, and smoked more frequently (Table 1).

**Table 1:** Description of characteristics of included women; overall and stratified by diagnosis of CIN2+

Characteristic	Total	Women with CIN2+	Women without CIN2+
N of participants in dataset regarding baseline sociodemographic characteristics Lifelines (%) ^a^	6237	195 (3.1)	6042 (96.9)
Median age at completing baseline questionnaire Lifelines in years (IQR)	30 (10)	27 (9)	30 (10)
Median year of completing baseline questionnaire Lifelines (IQR)	2012 (2)	2012 (2)	2012 (2)
Net income per month, N (%)			
Less than €1500	1747 (28.0)	76 (39.0)	1671 (27.7)
€1500 - €3000	2228 (35.7)	67 (34.4)	2161 (35.8)
More than €3000	1373 (22.0)	31 (15.9)	1342 (22.2)
Unknown, uncertain or not willing to answer	889 (14.2)	21 (10.7)	868 (14.3)
Educational level^b^, N (%)			
Low	1100 (17.6)	45 (23.1)	1055 (17.5)
Middle	2355 (37.8)	79 (40.5)	2276 (37.7)
High	2770 (44.4)	~ 70 (~ 35.9)	~ 2700 (~ 44.7)
Missing	12 (0.2)	< 10 (~ 0.5)^c^	~ 10 (~ 0.1)
Marital status, N (%)			
With a partner	4369 (70.0)	112 (57.4)	4257 (70.5)
Single	890 (14.3)	40 (20.5)	850 (14.1)
Other	260 (4.2)	13 (6.7)	247 (4.1)
Missing	718 (11.5)	30 (15.4)	688 (11.4)
Hormonal contraceptive use			
N of women who ever used hormonal contraceptives (%)	5602 (89.8)	176 (90.3)	5426 (89.8)
N missing (%)	453 (7.3)	~ 15 (~ 7.7)	~ 438 (~ 7.2)
Median number of years using hormonal contraceptives in last 10 years (IQR)	6.25 (6.0)	7.0 (5.2)	6.2 (6.0)
N missing (%)	196 (3.5)	< 10 (~ 2.8)^c^	~ 190 (~ 3.5)
Median number of pregnancies (IQR)			
For total group	1 (2)	0 (2)	1 (2)
For women at least one time pregnant (*N* = 3233)	2 (2)	2 (2)	2 (1)
N of women born after 1992 (%)	539 (8.6)	20 (10.3)	519 (8.6)
N of participants in dataset regarding childbirth data (%) ^a^	6209	192 (3.1)	6017 (96.9)
At least one childbirth, N (%)	3047 (49.1)	69 (35.9)	2978 (49.5)
Mean age at first child (sd)	26.6 (3.8)	25.8 (4.7)	26.6 (3.7)
Mean number of childbirths before the age of 30 (sd)	1.27 (0.9)	1.20 (0.9)	1.27 (0.9)
N of participants in dataset regarding smoking (%) ^a^	6276	197 (3.1)	6079 (96.0)
Smoking status, N (%)			
Never smoker	3109 (49.5)	62 (31.5)	3047 (50.1)
Ex-smoker	1119 (17.8)	33 (16.8)	1086 (17.9)
Current smoker	1564 (24.9)	81 (41.1)	1483 (24.4)
Missing	484 (7.7)	21 (10.7)	463 (7.6)

^a^ Participant numbers vary due to differences in data availability across Lifelines questionnaires

^b^ Marital status category “other” included women who indicated being widow, divorced or “other”

^c^ Exact frequencies under *N* = 10 are not displayed to ensure privacy for Lifelines participants

### Cervical test records characteristics

**Fig. 2:**
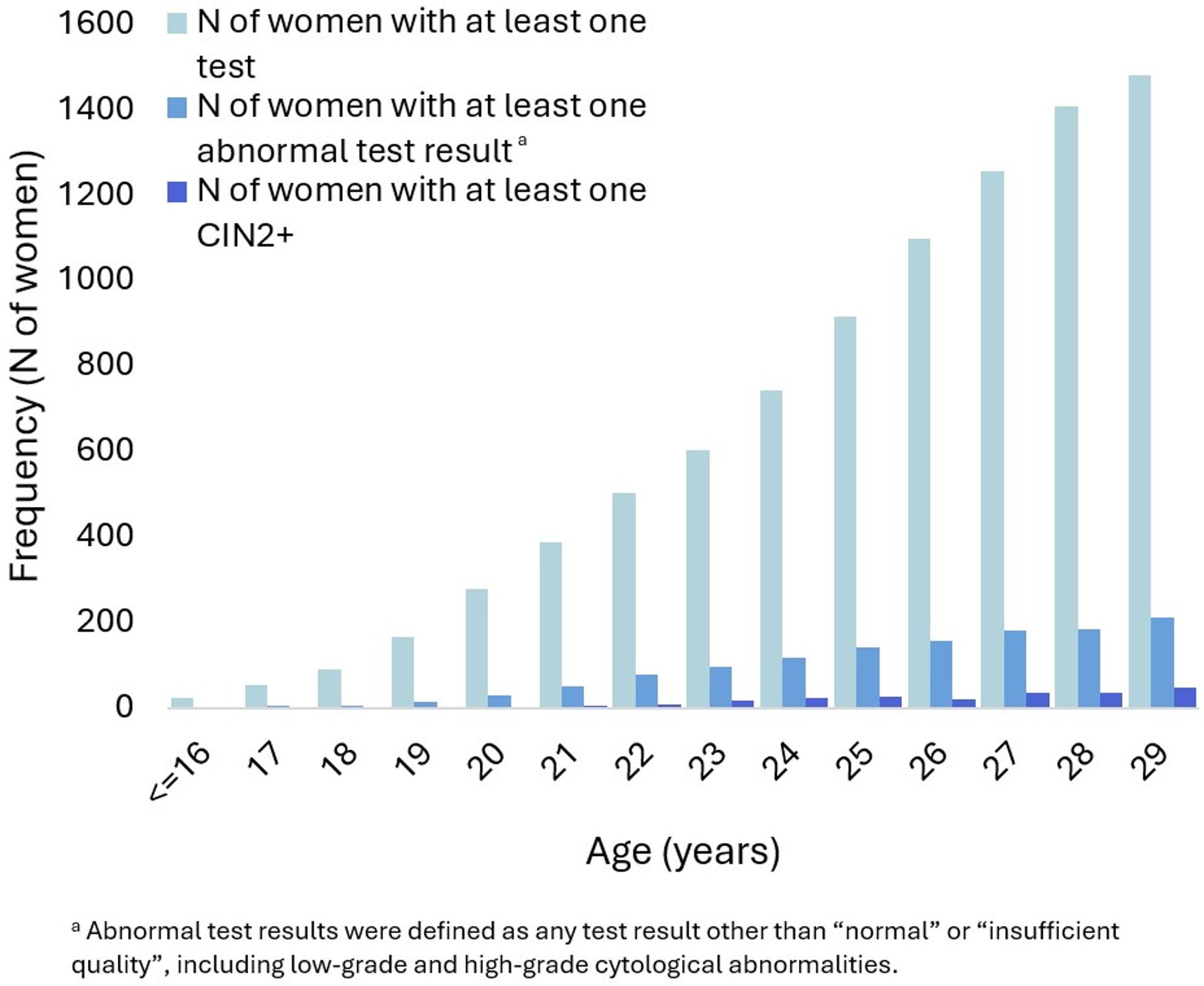
Frequency of cervical tests and outcomes for women below PBS age, per age

Characteristics of cervical test records registered in Palga of women below the PBS age can be found in Table 2. Most first cervical tests were performed by a GP (*N* = 3787, 60.1%). The median year of the first test record was later for women with a diagnosis of CIN2 + as compared to women without a diagnosis of CIN2+. The median age at first histologically confirmed CIN2 + diagnosis was 27 years (range 19–29). The most commonly diagnosed CIN2 + lesion was CIN3 (*N* = 115, 58.1%), followed by CIN2 (~ 36.9%). Less than 1% was diagnosed with a cervical carcinoma. The percentage of women with at least one abnormal test result below the PBS age varied between 5.5% and 15.9% across ages < 16 up until 29. The percentage of women with at least one CIN2 + increased from 0.0% at the age ≤ 16 until 3.2% at the age of 29 (Fig. 2).

**Table 2:** Characteristics of cervical test records below PBS age; overall and stratified by diagnosis of CIN2+

Characteristic	Total	Women with CIN2+	Women without CIN2+
Women with cervical test record in Palga below the PBS age, N (%)	6301	198 (3.1)	6103 (96.9)
Median age at first test record, age in years (IQR)	26 (5)	25 (4)	26 (5)
Median year of first test record, year (IQR)	2006 (9)	2008 (9.25)	2006 (10)
Provider of first test record, N (%)			
General practitioner^a^	3787 (60.1)	129 (65.2)	3658 (59.9)
Gynaecologist	2092 (33.2)	53 (26.8)	2039 (33.4)
Unknown or missing	422 (6.7)	16 (8.1)	406 (6.7)
Median age at first CIN2 + diagnosis, age in years (range)		27 (19–29)	
Most severe diagnosis, N (%)^c^			
Normal	5317 (84.4)		5317 (87.2)
Abnormal, less than CIN2	695 (11.0)		695 (11.4)
Abnormal, CIN2+	198 (3.1)	198 (100.0)	
CIN2	~ 73 (~ 1.2)	~ 73 (~ 36.9)	
CIN3	115 (1.8)	115 (58.1)	
Cervical carcinoma	~ 4–10 (< 1.0) ^b^	~ 4–10 (< 5.0)^b^	
Insufficient quality or no diagnosis	91 (1.4)		91 (1.5)

^a^ Registered in Palga as “other than gynaecologist”

^b^ Exact frequencies under *N* = 10 are not displayed to ensure privacy for Lifelines participants

^c^ The most severe diagnosis per woman at any age below the PBS age was classified according to the standard Palga order based on histology, or cytology if histology was unavailable. Abnormal test results were defined as any test result other than “normal” or “insufficient quality”, including low-grade and high-grade cytological abnormalities

### Association between sociodemographic characteristics and cervical intraepithelial neoplasia grade 2 and higher

Women with a low educational level more often had a diagnosis of CIN2+ (OR adjusted for age and year (hereafter: aOR) 1.7 (95%CI 1.1–2.4)) as compared to women with a high educational level. Ex-smokers and current smokers had significantly higher odds of a diagnosis of CIN2 + compared to never smokers (aOR 1.8, 95%CI 1.2–2.9 and aOR 2.7, 95%CI 1.9–3.8, respectively) (Table 3). Both sensitivity analyses, as shown in Additional File 1, showed comparable results.

**Table 3:** Association between sociodemographic characteristics and CIN2 + diagnosis for women below PBS age

Characteristics	aOR^a^ (95% CI)		*p*-value
Income			
>3000 (ref)	-		-
1500–3000	1.2 (0.8–1.9)		0.32
<1500	1.4 (0.9–2.3)		0.14
Educational level			
High (ref)	-		-
Middle	1.4 (1.0-1.9)		0.06
Low	1.7 (1.1–2.4)		0.009*
Marital status			
With a partner (ref)	-		-
Single	1.4 (1.0-2.1)		0.08
Ever use of hormonal contraceptives			
No (ref)	-		-
Yes	1.6 (0.6–4.4)		0.36
Years of hormonal contraceptive use in past 10 years	1.0 (1.0-1.1)		0.12
Number of pregnancies	1.0 (0.8–1.1)		0.68
Given childbirth			
No (ref)	-		-
Yes	0.8 (0.5–1.2)		0.23
Age at first child	1.0 (0.9–1.1)		0.67
Number of childbirths under age of 30	0.8 (0.6–1.1)		0.23
Smoking status			
Never smoker (ref)	-		-
Ex smoker	1.8 (1.2–2.9)		0.006*
Current smoker	2.7 (1.9–3.8)		< 0.001*

^a^aOR = Odds Ratios of separate regression models, adjusted for age at completing baseline questionnaire and year of completing in baseline questionnaire

* Significant with α = 0.05

### Cervical intraepithelial neoplasia grade 2 and higher in women at eligible age for participation in first round of population-based screening

The percentage of detected CIN2 + in women at the eligible ages for participation in the first round of PBS was 2.2% for women with a PBS record and 3.0% for women without a PBS record, as presented in Additional File 2.

## Conclusions

### Summary

In our cohort, we found that 3.1% of women tested and/or referred by the GP before reaching the eligible age for participation in the Dutch PBS for cervical cancer were diagnosed with clinically relevant (pre)malignant cervical disease. The odds for clinically relevant (pre)malignant disease were significantly higher for women with low educational level and women who (used to) smoke.

### Strengths and limitations

Strengths of this research rely in the data sources; Lifelines allowed us to obtain routinely, systematically collected data for a large number of women representative for the north of the Netherlands, and the data obtained from Palga provides unique and reliable nationwide coverage of 100% of pathology records in the Netherlands [12, 14].

A limitation of this study is that we were not able to evaluate the occurrence of (pre)malignant cervical disease below PBS age in (a)symptomatic women that were not tested. However, testing is recommended by the guidelines in case of a large variety of symptoms suggestive of the presence (pre)malignant cervical disease [10, 11]. Also, the number of CIN2 + diagnoses in the first round of the PBS, which is in line with nationwide percentages, does not indicate a particularly high occurrence in asymptomatic women below PBS age [17]. Secondly, our data lacked information on hrHPV vaccination status. However, its impact on our findings is presumed minimal due to the small number of women included from eligible birth cohorts, and low initial uptake [18]. Additionally, due to its relatively recent introduction, too few HPV test results were available to stratify by hrHPV infection status. This research however focuses on aiding a GP’s clinical decision making prior to diagnostic testing; hrHPV status will also not be known at that moment. Furthermore, cervical tests were assumed to have been performed due to symptoms suggestive of the presence of (pre)malignant cervical disease, but may also have been conducted for reasons such as abnormalities observed during screening for sexually transmitted diseases or the placement of an intra-uterine device. However, the registration of indications for performing these tests appeared to be incomplete for our cohort. Moreover, conducting this research within the context of the Dutch screening programme is primarily limited to countries with a similar screening contexts (i.e. starting at the age 30). Lastly, collecting sociodemographic characteristics only after CIN2 + diagnosis for part of the cohort, may have introduced information bias. However, these characteristics are expected to be relatively stable in the short term, and the sensitivity analysis restricted to women who completed the questionnaire before diagnosis yielded similar results.

### Comparison with existing literature

The prevalence of (pre)malignant cervical disease before the first round of PBS aligns with prior evidence. The process from hrHPV infection to CIN development usually takes several years, and cervical cancer risk is low in the first years after sexual debut, i.e., potential hrHPV exposure [19, 20]. Also, spontaneous regression rates of CIN are high in young women [16, 19, 21, 22]. Van der Waal et al. reported in a Dutch study a CIN2 + prevalence of 5.8% in women aged 18–29 at baseline, however also including (PBS-detected) CIN2 + diagnoses after age 30 [23]. Other European studies report higher CIN2 + rates up to age 30, though comparisons are limited due to inclusion of populations with lower starting ages (age 20/25) of the PBS [24, 25].

Although we did not focus on causality, the observed associations may partially reflect causal relationships. Smoking is known to enhance the risk of (pre)malignant cervical disease, potentially independently of hrHPV status [7, 26, 27]. The association with educational level may be explained by patient factors such as sexual behaviour and health consciousness [28]. Van der Waal et al. also found lower odds or CIN2 + for higher educational levels in the Netherlands, but reported no association between smoking and CIN2+ [23]. This difference might possibly be due to their inclusion of CIN2 + after age 30, a smaller sample size and/or timing of smoking assessment. International studies align with the found association on smoking, but show mixed results for other sociodemographic factors. However, many of these studies were conducted in low- and middle income countries, where general sociodemographic characteristics may differ substantially from those in the Netherlands [29–36].

### Implications for the general practice

Among included women, 8.7% received (a referral for) cervical testing from the GP before the eligible age for participation in the first round of the Dutch PBS. Around 1 in 30 (3.1%) of these women were found to have clinically relevant (pre)malignant cervical disease. When a woman below PBS age presents with symptoms potentially suggestive of (pre)malignant cervical disease, we recommend that GPs consider the higher odds in those with a history of smoking and/or low educational level – while aligning their clinical decision making with existing guidelines [10, 11].

### Evidence on indication of population-based screening under the age of 30

The prevalence of (pre)malignant cervical disease, including cervical carcinoma prior to and during the first PBS round, may suggest that the starting age of 30 for the PBS has been appropriate for this cohort. However, it should be kept in mind that the prevalence in untested and unscreened women is unknown, especially as early-stage disease is often asymptomatic [7]. The Dutch Cancer Registry shows that in 2022, 3% of cervical cancer cases was diagnosed under 30 [37]. European studies on cohorts screened under 30 report substantial CIN2/3 prevalences, but also a steep decrease in incidence in recent years, possibly due to the introduction of the hrHPV vaccination [24, 38]. Disadvantages of the PBS such as overdiagnosis and overtreatment (in turn e.g. possibly leading to an increased risk of adverse obstetric outcomes), should be weighed against screening effectivity and advantages [39]. Given the low cervical cancer mortality among Dutch women under the age of 30 and the high spontaneous regression rates, Van der Aa et al. argued that the disadvantages do not outweigh the benefits in this age group. They argue that, instead, efforts should focus on increasing the PBS participation rates for the age groups currently included in the Dutch PBS [40]. It is also important to consider that recent developments in cervical cancer prevention, such as the introduction of the hrHPV vaccination and self-sampling, will likely require a re-evaluation of current screening strategies, both for women within and the current target age groups.

### Future perspectives

Recent studies show the significant impact of hrHPV vaccination, with fewer cervical abnormalities in vaccinated women [41, 42]. As younger cohorts will increasingly include women who have been eligible for hrHPV vaccination, incorporating vaccination status should be considered essential in research aimed to guide the GPs clinical decision making for young women presenting with symptoms that are suggestive of (pre)malignant cervical disease.

## Conclusions

Within women tested and/or referred by the GP before the eligible age for the Dutch cervical cancer PBS, 3.1% was diagnosed with CIN2+. Higher odds of CIN2 + for women with a low educational level and women who (used to) smoke should be taken along in clinical decision making towards testing and referral by general practitioners.

## Supplementary Information

Additional File 1.

Additional File 2.

## Data Availability

The data that support the findings of this study are available from Lifelines and Palga but restrictions apply to the availability of these data, which were used under license for the current study, and so are not publicly available. Data are however available from the authors upon reasonable request and with permission of Lifelines and Palga.

## Abbreviations

CIN2+: high-grade cervical intra-epithelial neoplasia
GP: general practitioner
(hr)HPV: (high-risk) human papillomavirus
PBS: population-based screening
