# Supplementary material for: Young women with (pre)malignant cervical disease in northern Netherlands: what characterises them – A population-based cohort study
Source: BMC Womens Health. 2026 Jun 11;26:404. doi: 10.1186/s12905-026-04598-6 (PMC13479446; doi:10.1186/s12905-026-04598-6)
Supplement: Supplementary file 1 [file 12905_2026_4598_MOESM1_ESM.docx]

**Additional File 1. Sensitivity analyses**

| **Characteristics** | **First sensitivity analysis^a^** | | **Second sensitivity analysis^b^** | |  |
| --- | --- | --- | --- | --- | --- |
|  | **aOR^c^ (95% CI)** | **p-value** | **aOR^c^ (95% CI)** | **p-value** |  |
| **Income** |  |  |  |  |  |
| >3000 (ref) | - | - | - | - |  |
| 1500-3000 | 5.9 (0.8-45.3) | 0.087 | 1.4 (0.8-2.4) | 0.242 |  |
| <1500 | 8.9 (1.2-68.3) | 0.035* | 1.5 (0.8-2.8) | 0.162 |  |
| **Educational attainment** |  |  |  |  |  |
| High (ref) | - | - | - | - |  |
| Middle | 1.5 (0.9-2.6) | 0.156 | 1.4 (0.9-2.1) | 0.133 |  |
| Low | 1.3 (0.7-2.5) | 0.395 | 2.2 (1.4-3.5) | 0.001* |  |
| **Marital status** |  |  |  |  |  |
| With a partner (ref) | - | - | - | - |  |
| Single | 1.7 (0.9-3.0) | 0.085 | 1.4 (0.9-2.4) | 0.152 |  |
| **Ever use of hormonal contraceptives** |  |  |  |  |  |
| No (ref) | - | - | - | - |  |
| Yes | 2.0 (0.5-8.4) | 0.343 | 1.9 (0.5-7.8) | 0.370 |  |
| **Years of hormonal contraceptive use** **in past 10 years** | 1.1 (1.0-1.1) | 0.038* | 1.0 (1.0-1.1) | 0.051* |  |
| **Number of pregnancies** | 1.1 (0.7-1.5) | 0.775 | 1.0 (0.8-1.2) | 0.733 |  |
| **Given childbirth** |  |  |  |  |  |
| No (ref) | - | - | - | - |  |
| Yes | 1.2 (0.5-2.8) | 0.690 | 0.9 (0.5-1.4) | 0.513 |  |
| **Age at first child** | 0.8 (0.6-1.0) | 0.055 | 1.0 (0.9-1.1) | 0.809 |  |
| **Number of childbirths under age of 30** | 1.1 (0.3-3.4) | 0.903 | 0.7 (0.5-1.1) | 0.119 |  |
| **Smoking status** |  |  |  |  |  |
| Never smoker (ref) | - | - | - | - |  |
| Ex smoker | 1.5 (0.6-4.1) | 0.419 | 1.8 (1.0-3.1) | 0.056 |  |
| Current smoker | 3.0 (1.8-5.1) | 0.000* | 3.1 (2.0-4.8) | 0.000* |  |
| ^a^ Including only women diagnosed with cervical intraepithelial neoplasia grade 2, cervical intraepithelial neoplasia grade 3 or cervical carcinoma (CIN2+) who completed in the baseline questionnaire before the diagnosis (N = 73 CIN2+, 6042 non CIN2+, total N = 6115), to exclude effect altering by changing behaviour after receiving the diagnosis | | | | |  |
| ^b^ Defining cases as CIN3+ women (and comparing them to non CIN3+ women) (N= 118 CIN3+, N=6119 non CIN3+, total N= 6237) | | | | |  |
| ^c^  aOR = Odds Ratios of separate logistic regression models, adjusted for age at completing baseline questionnaire and year of completing baseline questionnaire | | | | |  |
| * Significant with α = 0.05 | | | | |  |
