# Supplementary material for: Young women with (pre)malignant cervical disease in northern Netherlands: what characterises them – A population-based cohort study
Source: BMC Womens Health. 2026 Jun 11;26:404. doi: 10.1186/s12905-026-04598-6 (PMC13479446; doi:10.1186/s12905-026-04598-6)
Supplement: Supplementary file 2 [file 12905_2026_4598_MOESM2_ESM.docx]

### **Additional File 2. Results of tests of women at eligible ages for the first round of population-based screening (ages 30-34)**

|  | **Women with PBS record^a^** | **Women without PBS record** |
| --- | --- | --- |
| **Number of women with at least one test performed** | 19956 | 3268 |
| **Number of women with abnormal first test results (%)** | 1190 (6.0) | 273 (8.4) |
| **Total number of CIN2+ (%)** | 446 (2.2) | 97 (3.0) |
| Number of CIN2+ screen-detected^b^ (%) | 385 (86.3) |  |
| Number of CIN2+ clinically detected^c^ (%) | 61 (13.7) | 97 (100.0) |
| ^a^ Women were considered to have participated in the first round of PBS if they had a first population-based screening (PBS) record between the ages of 29 and 33. Diagnoses up to the age of 34 were included if these were preceded by a PBS record before the age of 34. However, diagnoses at the age of 34 were not taken along if they were preceded by a first PBS record at 34, as that could also have been in the context of the second screening round. | | |
| ^b^ A diagnosis of CIN2+ was registered as screen-detected when it was preceded by an abnormal PBS test result and advice for follow-up. | | |
| ^c^ A diagnosis of CIN2+ was registered as clinically detected for PBS attendants when the PBS result was normal and/or no follow-up advice was given based on the PBS result. | | |
